# A Changed Gut Microbiota Diversity Is Associated With Metabolic Improvements After Duodenal Mucosal Resurfacing With Glucagon-Like-Peptide-1 Receptor Agonist in Type 2 Diabetes in a Pilot Study

**DOI:** 10.3389/fcdhc.2022.856661

**Published:** 2022-07-05

**Authors:** Suzanne Meiring, Annieke C. G. van Baar, Nikolaj Sørensen, Frits Holleman, Maarten R. Soeters, Max Nieuwdorp, Jacques J. G. H. M. Bergman

**Affiliations:** ^1^ Gastroenterology and Hepatology, Amsterdam University Medical Centres, Amsterdam, Netherlands; ^2^ Scientific Operations Clinical Microbiomics, Copenhagen, Denmark; ^3^ Internal Medicine, Amsterdam University Medical Centres, Amsterdam, Netherlands; ^4^ Endocrinology, Amsterdam University Medical Centres, Amsterdam, Netherlands; ^5^ Internal and Vascular Medicine, Amsterdam University Medical Centres, Amsterdam, Netherlands

**Keywords:** duodenal mucosal resurfacing, gut microbiota, GLP-1RA, diabetes type 2, endoscopic, DMR, microbiota diversity

## Abstract

**Introduction:**

The gut microbiota influences and interacts with the host metabolism through effects on nutrient metabolism and digestion. Duodenal Mucosal Resurfacing (DMR) is a novel endoscopic procedure involving duodenal mucosal ablation by the use of hydrothermal energy. DMR, when combined with a glucagon-like peptide-1 receptor agonist (GLP-1RA), resulted in discontinuation of exogenous insulin treatment in 69% of patients with insulin dependent type 2 diabetes mellitus (T2DM) in the INSPIRE study. These patients also experienced improved glycaemic control and metabolic health. We thus investigated if these clinical effects were associated with a change in gut microbiota alpha and beta diversity.

**Methods:**

Faecal samples from the 16 patients were obtained for Illumina shotgun sequencing at baseline and 3 months after DMR. We assessed alpha and beta diversity of the gut microbiota in these samples and analysed its correlations with changes in HbA1c, body weight, and liver MRI proton density fat fraction (PDFF).

**Results:**

HbA1c correlated negatively with alpha diversity (*p*=0.011, rho: -0.62) whereas changes in PDFF correlated significantly with beta diversity (*p*=0.036, rho: 0.55) 3 months after initiation of the combined intervention. These correlations with metabolic parameters were observed despite finding no change in gut microbiota diversity at 3 months post DMR.

**Discussion:**

The correlation between gut microbiota richness (alpha diversity) and HbA1c as well as the change in PDFF and changed microbiota composition (beta diversity) suggests that changed gut microbiota diversity is associated with metabolic improvements after DMR in combination with glucagon-like-peptide-1 receptor agonist in type 2 diabetes. Larger controlled studies are however needed to find causal links between DMR with GLP-1RA, the gut microbiota, and improvements in metabolic health.

## Introduction

Diabetes mellitus is nowadays one of the most important public health challenges. About 1 in 11 adults worldwide has type 2 diabetes mellitus (T2DM) and its prevalence is still rising (1). Many patients with T2DM eventually require insulin therapy to maintain glycaemic control, but this symptomatic therapy does not address insulin resistance, the root phenomenon of T2DM, and can contribute to a deterioration of metabolic health (2). In this regard, exposure to a Western diet, rich in refined sugars and saturated fats, can lead to changes in gut microbial composition and physiology, which in turn are linked to development of T2DM (3). In this regard, the gut microbiota interacts and influences the host metabolism through effects on nutrient metabolism and digestion, bile acid synthesis, energy metabolism, gut barrier function, the immune system, and xenobiotic metabolism. The interest for the role of the gut microbiota in the aetiology and potential treatment of obesity and T2DM has therefore grown over the last decades (3, 4). Indeed there is consensus that T2DM patients are characterized by an overall lower gut microbiota diversity compared to healthy individuals (5).

Bariatric surgery is the most effective treatment for T2DM with a high long-term remission rate (6, 7) and major improvements in metabolic and cardiovascular health. Glycaemic improvements (6–9) occur overnight after surgery, even before any significant weight loss has occurred (10–12). Multiple studies have found an overall increase in gut microbial diversity in patients with T2DM after bariatric surgery (13). Additionally, metabolic improvements in patients receiving a duodenal jejunal bypass liner are accompanied by an increase in microbial diversity (14). It is therefore hypothesized that changes in gut microbiota composition play a regulating role in the positive glycaemic and metabolic effects after interventions involving the small bowel.

Duodenal Mucosal Resurfacing (DMR) is a minimally invasive endoscopic procedure that administers hydrothermal energy to ablate the duodenal mucosa (15). Data from animal models and human studies suggest that a single DMR elicits improvements in insulin sensitivity, similar to the metabolic improvements seen after bariatric surgery, albeit in a lesser extent (16). The physiological mechanism underlying the efficacy of DMR has yet to be elucidated, but we hypothesize that changes in the gut microbiota composition play a role in this effect, in line with other interventions involving the small bowel.

We recently published on the effect of a combination treatment of DMR and glucagon-like peptide-1 receptor agonist (GLP-1RA) administration in a pilot study of 16 patients with T2DM, treated with insulin and found an overall improvement in glycaemic and metabolic endpoints, leading to a discontinuation of insulin treatment in 69% of the patients at 6 months. In the current spin-off study, we explored whether a change in gut microbiota diversity and composition was seen after initiation of the DMR and GLP-1RA combination treatment in these patients. We also explored whether these changes were associated with improved glycaemic and metabolic parameters, including liver fat fraction, and if change in specific bacterial species were seen.

## Materials and Methods

### Study Design

The original pilot study was a single-centre, single-arm, prospective, open-label clinical study that investigated the effect of a single DMR procedure combined with GLP-1RA (liraglutide), in patients with T2DM, treated with insulin on glycaemic and metabolic health. The study protocol was approved by the medical ethics committee of the Amsterdam University Medical Center. The study was conducted in accordance with ICH Good Clinical Practice Guidelines and the Declaration of Helsinki. The study is registered under EudraCT number 2017-00349-30 at Clinicaltrialsregister.eu. The main clinical outcomes of this study have been reported previously. This report concerns a spin-off study, investigating the association between metabolic improvements and changes in gut microbiota after initiation of the combination treatment of DMR and GLP-1RA.

### Clinical Study Summary

We included 16 patients with T2DM using long-acting insulin, aged 28-75 years, with a body mass index of 24-40 kg/m2, and an HbA1c ≤ 8.0% (64 mmol/mol). The list of inclusion and exclusion criteria can be found in **Supplementary Table 1**. Patients were primarily recruited *via* the general practitioner. Written informed consent was obtained from all patients. Endoscopic DMR was performed under deep sedation with propofol by a single endoscopist (JJGHM) with experience in endoscopic DMR procedures (15, 16). The DMR procedure involved circumferential hydrothermal ablation of the duodenal mucosa using an over-the-guidewire catheter, as described previously (15, 16). Exogenous insulin administration was discontinued the day before the DMR procedure according to the study protocol. Patients were instructed by a dietician to adhere to a tailored isocaloric 2-week post-procedural diet (i.e. gradual transition from liquid to solid food) to allow adequate healing of the duodenal mucosa. Patients began with self-administration of subcutaneous GLP-1RA, liraglutide (Victoza^®^, Novo Nordisk A/S) once daily at a standard dosage of 0.6 mg/day that was gradually increased to 1.8mg/day, as registered for treatment of T2D, after finishing the post-procedural diet. Standard mild nutritional counselling and lifestyle education were provided before DMR and during follow-up (17). All oral glucose-lowering medications were continued in the same dosage throughout the study. Insulin was reintroduced (and liraglutide discontinued) when HbA1c was >9.5% at 3 months follow-up, >7.5% at 6, 12, 18 months follow-up or if self-measured fasting glucose was >270 mg/dl on 3 consecutive days. The primary endpoint of this pilot study was the percentage of patients free of exogenous insulin therapy with adequate glycaemic control, defined as HbA1c ≤ 7.5% at the six-month follow-up (responders). Secondary endpoints were changes compared to baseline in glycaemic parameters during follow-up (HbA1c, HOMA-IR, FPG) and in metabolic parameters (BMI, ALT and PDFF) to evaluate additional metabolic benefits of the combination treatment applied in this pilot study. These primary and secondary endpoints have been published previously (17). This additional report focusses on the changes in gut microbiota composition and diversity.

### Data Collection

#### Clinical and Anthropometric Evaluations

At baseline and at 6 months follow-up body weight and HbA1c levels were measured and magnetic resonance imaging (MRI; model clinical 3 Tesla scanner, Achieva, Philips) was used to calculate the liver proton density fat fraction (PDFF).

#### Collection of Faecal Samples

Patients were instructed to collect a morning faeces sample prior to the visit at baseline (1 week before DMR) and 3 months after DMR. The sample was collected by the patient using gloves, put into a faeces collection tube and directly frozen at -20°C in their freezer at home. Once arrived at the hospital, all samples were immediately stored at -80°C until the actual analyses.

### Laboratory Assessments and Gut Microbiota Analysis

#### Dna Extraction

Approximately 0.2 g of faecal material was used per extraction. DNA was extracted from samples using NucleoSpin^®^ 96 Soil (Macherey-Nagel). Bead beating was done on a Vortex-Genie 2 horizontally for 5 min. A minimum of one negative control was included per batch of samples from the DNA extraction and throughout the laboratory process (including sequencing). A ZymoBIOMICS™ Microbial Community Standard (Zymo Research) was also included in the analysis.

#### Shotgun Sequencing

Faecal microbiota sequencing was done at Clinical Microbiomics (Copenhagen, Denmark). Before sequencing, the quality of the DNA extractions was evaluated using agarose gel electrophoresis and the quantity by Qubit 2.0 fluorometer quantitation. The genomic DNA was randomly sheared into fragments of around 350 bp. The fragmented DNA was used for library construction using NEBNext Ultra Library Prep Kit for Illumina (New England Biolabs). The prepared DNA libraries were evaluated using Qubit 2.0 fluorometer quantitation and Agilent 2100 Bioanalyzer for the fragment size distribution. Quantitative real-time PCR (qPCR) was used to determine the concentration of the final library before sequencing. The library was sequenced using 2 × 150 bp paired-end sequencing on an Illumina platform.

#### Gene Catalogue and Metagenomic Species Definitions

As a reference gene catalogue, we used the Clinical Microbiomics Human Gut 22M gene catalogue (22,459,186 genes), which was created from >5000 deep-sequenced human gut specimens. For MGS abundance profiling, we used the Clinical Microbiomics HGMGS v.2.3 set of 1273 metagenomic species (MGS), which have highly coherent abundance and base composition in a set of 1776 independent reference human gut samples. The approach is based on the metagenomic species concept (18). To taxonomically annotate the MGSs, we blasted all the catalogue genes to the NCBI RefSeq genome database (2018-10-01) and used different levels of similarity (95, 95, 85, 75, 65, 55, 50 and 45% for subspecies, species, genus, family, order, class, phylum, superkingdom, respectively) to annotate at the various taxonomic levels and requiring a minimum of 80% sequence coverage. We calculated the percentage of genes of each MGS that mapped to each species and assigned species level taxonomy to a MGS if > 75% of its genes could be annotated to a given species. For genus, family, order, class and phylum, we used 60, 50, 40, 30 and 25% consistency, respectively. Furthermore, at species and at genus level, we allowed a maximum of 10% of the genes belonging to a MGS to be annotated to an alternative taxon. Finally, we also used checkM to annotate the MGSs, and updated our annotations with checkM results in cases where checkM could annotate an MGS at higher resolution (lower taxonomic rank).

#### Sequencing Data Pre-Processing

Quality control of raw FASTQ files was performed using KneadData (v. 0.6.1) to remove low-quality bases and reads derived from the host genome.

#### MGS Relative Abundance Calculation

For each MGS, the signature gene set was defined as the 100 genes optimized for accurate abundance profiling of the MGS. A table of MGS counts was created based on the total gene counts for the 100 signature genes of each MGS. However, an MGS was considered detected only if read pairs were mapped to at least three of its 100 signature genes; counts for MGSs that did not satisfy this criterion were set to zero. This threshold has in internal benchmarks resulted in 99.6% specificity.

#### Functional Annotation and Profiling

Emapper software (v. 1.0.3, HMM mode) was used to compare each gene in the gene catalog to the EggNOG (v. 4.5) orthologous groups database (http://eggnogdb.embl.de/), resulting in annotations for 65% of genes. These genes were then mapped from EggNOG to the Kyoto Encyclopedia of Genes and Genomes (KEGG) orthology (KO) database http://www.genome.jp/kegg/kegg1.html) using MOCAT2 lookup tables (http://mocat.embl.de/). Functional potential profiles based on KOs were calculated as the number of read pairs mapping to all genes annotated to a given KO, divided by the total number of mapped reads.

### Study Endpoints

The endpoints for this spin-off study were changes in gut microbiota alpha and beta diversity and abundance of bacterial species at 3 months after DMR compared to baseline. Additionally, we explored correlations between HbA1c, body weight and PDFF at 6 months and gut microbiota diversity at 3 months after DMR.

### Calculations and Statistical Analysis

Clinical parameters are expressed as medians with interquartile range [Q1-Q3]. Alpha diversity was calculated as richness (e.g. the number of MGSs observed in a sample). Beta diversity was calculated as dissimilarity in community composition between samples, using Bray-Curtis measurements. Bray-Curtis dissimilarity takes the abundance of species into account and can be 0–1, where 0 means that the two samples have identical compositions (they share all species at the same relative abundance), and 1 means that the two samples are completely different (they do not share any species). The abundance of MGSs in faecal samples before-treatment and after-treatment was compared using Wilcoxon signed-rank tests. Correlations between alpha diversity, beta diversity and abundance of MGSs and KOs were correlated with clinical variables (HbA1c, PDFF and body weight) using Spearman’s correlation. To correct for multiple hypothesis testing, false discovery rate was used for detecting false positives (using the conventional threshold of 0.10). Only MGSs with a prevalence of >20% in the investigated samples were included in the analyses.

## Results

### Patient Characteristics

All 16 enrolled patients underwent a successful DMR procedure. Patients were on average 61 years old, T2DM duration was 11 years, and patients used 31 units of insulin per day prior to DMR. All baseline characteristics can be found in **Supplementary Table 2**. At 6 months, 69% of patients (11/16) met the primary endpoint of the study: i.e. off insulin therapy with an HbA1c ≤ 7.5%. Patients also demonstrated significant improvements in secondary endpoints regarding metabolic health: fasting plasma glucose, body weight, total body fat and average MRI liver PDFF decreased significantly. Details on these clinical outcomes have been published previously (17).

### Shotgun Sequencing Results

**Figure 1:**
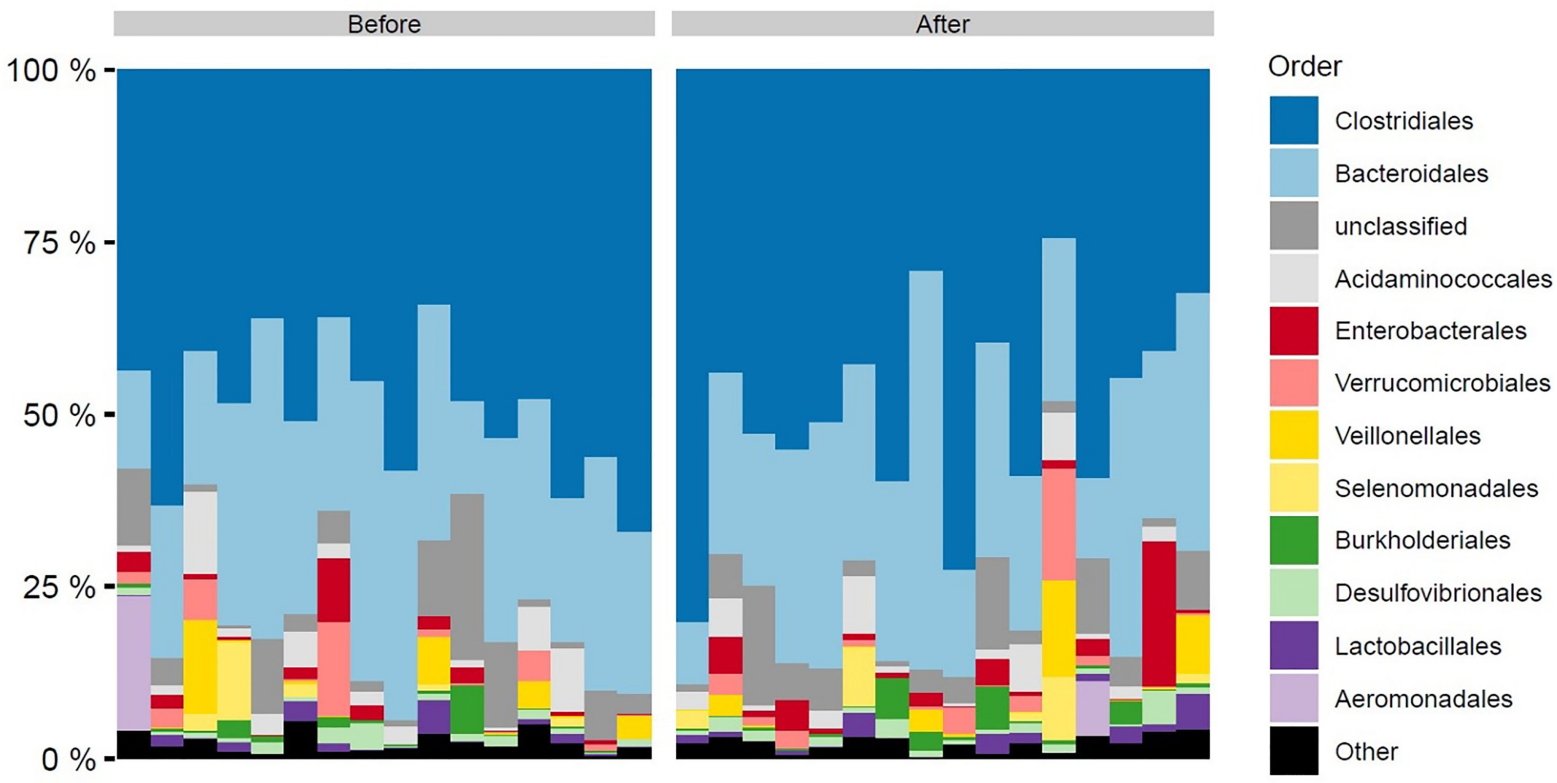
Order level taxonomic composition of faecal samples by time of sampling before and after DMR. Each bar represents a single patient sample. DMR, duodenal mucosal resurfacing.

Of the 15-23 million high-quality non-host reads generated for each of the 32 faecal samples, 42-89% mapped to the gene catalogue (18) and the Clinical Microbiomics human gut MGS database. Approximately 50% of the MGSs in all 32 faecal samples composed of *Clostridiales*, followed by *Bacteroidales* with ± 20%. Other taxa with high abundance were *Acidaminococcales, Enterobacterales, Verrucomicrobiales* and *Selenomonadeles*. Taxonomical profiles aggregated at order level are shown in **Figure 1**.

### Microbiota Diversity and MGS Abundance Did Not Change Significantly Upon Intervention

We did not find a change in faecal microbiota alpha diversity (richness) nor beta diversity (community composition) at 3 months follow-up compared to baseline. Moreover, we did not find significant changes in specific bacterial species.

### Microbiota Diversity Correlated to Clinical Parameters

**Figure 2:**
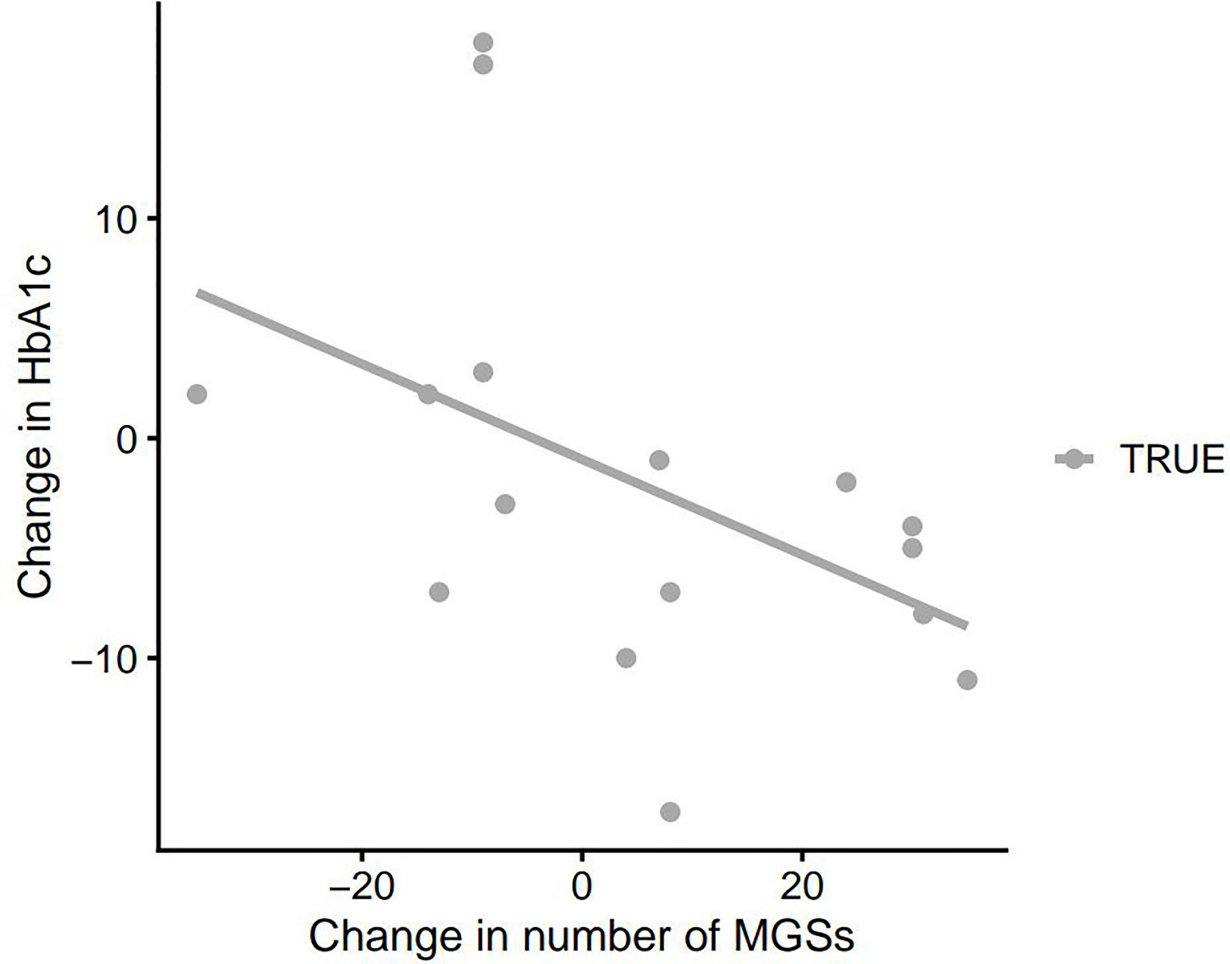
Change in HbA1c as a function of change in MGS richness in the faecal samples at baseline and 3 months after DMR. HbA1c, haemoglobin A1c; MGS, metagenomic species; DMR, duodenal mucosal resurfacing.

We observed an inverse correlation between the change in HbA1c and the change in gut microbiota alpha diversity (*p*=0.011, rho: -0.62). Thus a decrease in HbA1c was correlated with an increase in gut microbiota richness and vice versa (**Figure 2**). Moreover, changes in PDFF correlated significantly with gut microbiota beta diversity (community composition as determined by Bray-Curtis dissimilarity of species, *p*=0.036, rho: 0.55 and as determined by Bray-Curtis dissimilarity of KOs, *p*=0.035, rho: 0.55). Thus, a large change in liver fat content is associated with a large change in the gut microbiota diversity (**Figures 3A** and **3B**). No significant correlations between change in weight and change in gut microbiota diversity were found. Lastly, no significant correlations between changes in specific bacterial species and changes in other clinical variables were found.

**Figure 3A:**
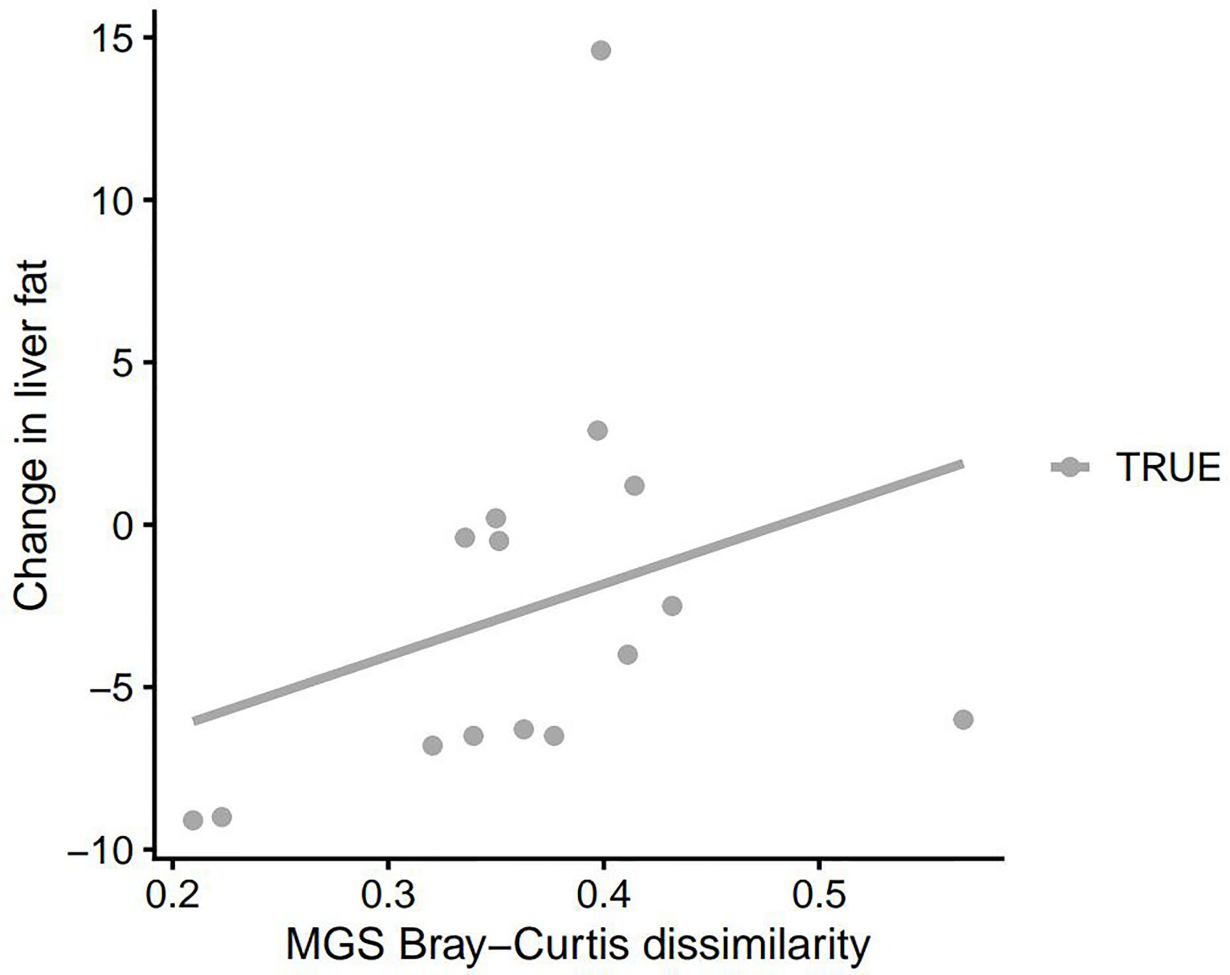
Change in PDFF as a function of MGS Bray-Curtis dissimilarity between faecal samples at baseline and 3 months after DMR. PDFF, proton density fat fraction; MGS, metagenomic species; DMR, duodenal mucosal resurfacing.

**Figure 3B:**
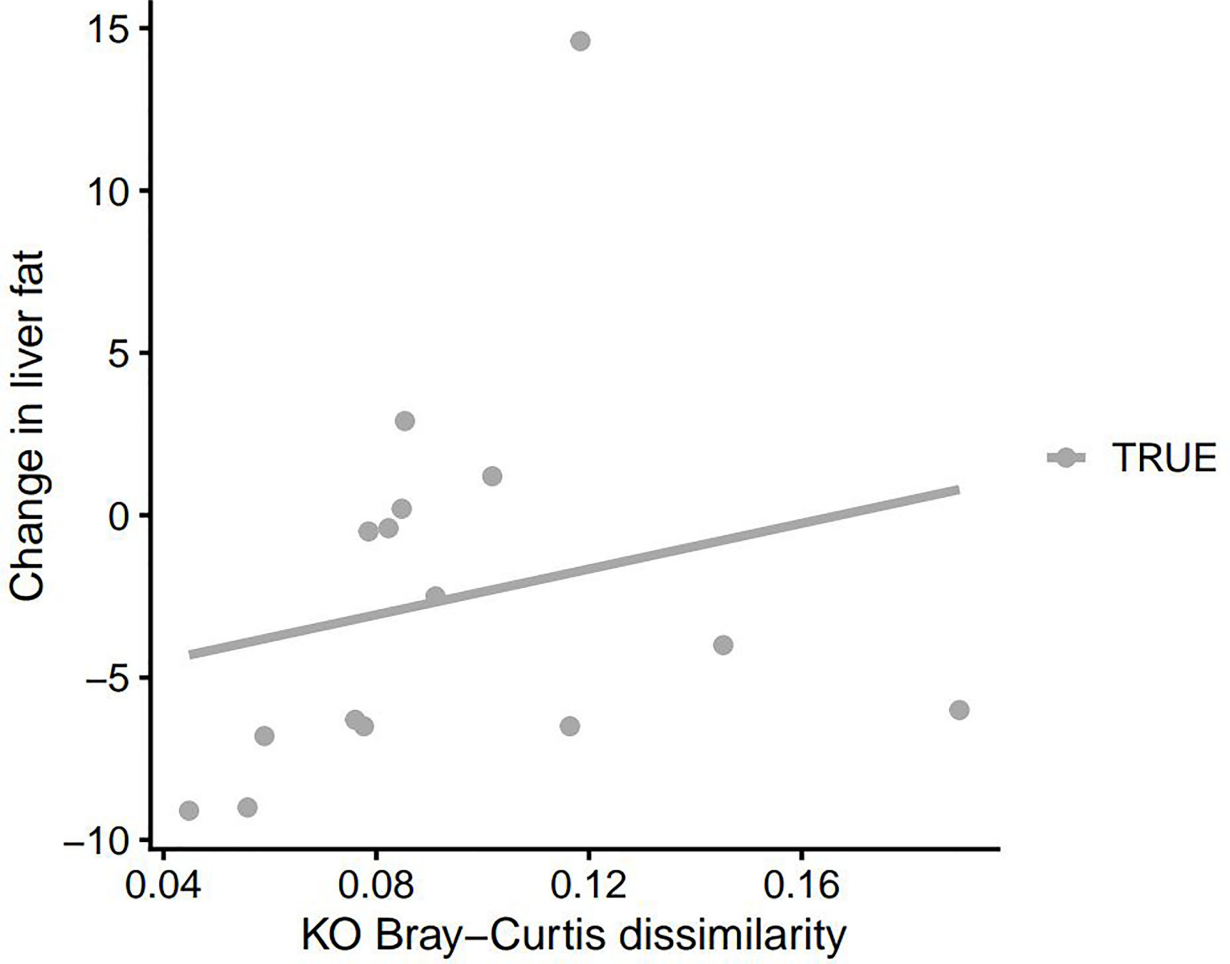
Change in PDFF as a function of KO Bray-Curtis dissimilarity between faecal samples at baseline and 3 months after DMR. PDFF, proton density fat fraction; KO, Kyoto Encyclopedia of Genes and Genomes (KEGG) orthology; DMR, duodenal mucosal resurfacing.

## Discussion

We found that after initiation of DMR with GLP-1RA, a decrease in HbA1c was associated with an increase in gut microbiota richness in faecal samples. Moreover, larger changes in PDFF at 3 months after DMR was associated with a more changed gut microbiota diversity. We did not find a significant change in abundance of any bacterial species after compared to before DMR. However, the sample size of our pilot study was limited and the background variability is known to be high in gut microbiota studies, so these results do not exclude the possibility that duodenal ablation is associated with relevant changes in the gut microbiota. This is currently the only clinical study in which faecal samples of patients were obtained for gut microbiota analysis to unravel the underlying insulin-sensitizing mechanism of the novel DMR procedure for type 2 diabetes.

Our first key finding was the negative correlation between HbA1c and gut microbiota richness. This means that improvement in glycaemic control was correlated with increased gut microbiota diversity in our patients with T2DM. These findings are supported by a recently published systematic review of other metabolic procedures, reporting increased gut microbiota diversity in patients with metabolic improvements following endoscopic and surgical metabolic procedures across several studies (3). However, we did not find increased diversity of the gut microbiota in our complete (n=16) population after the intervention compared to baseline. This might be explained by the fact that 5/16 of our patients did not develop glycaemic improvements post intervention, previously referred to as the *non-responders*. We can not exclude that this *non-responder* population affected the results in this rather small pilot population. Interestingly, in another spin-off report of this study, we observed increased postprandial unconjugated bile acids and increased secondary bile acids in the complete population (19). These findings suggest that the gut microbiota composition did change considerably in our patients, since bile acid deconjugation, conversion, and active uptake of bile acids are executed by gut bacteria. These findings are a step forward in elucidating the mechanism behind DMR and thereby possibly improving efficacy of the DMR procedure. It also adds to the evidence that gut microbiota play a major role in glycaemic and metabolic health, and that a changed gut microbiota is associated with insulin resistance in the context of metabolic syndrome. It is possible that change in gut microbiota diversity in the complete population could not be captured by the current available techniques, and our small sample size can be explanatory as well.

Our second key finding was that a large change in liver MRI PDFF was correlated with a large change in gut microbiota diversity. Clinical and pre-clinical studies show that T2DM and non-alcoholic fatty liver disease (NAFLD) often co-exist as they share a common pathway of adipose tissue dysfunction and hepatic insulin resistance (20–22). Microbiota-derived-compounds reach the liver *via* the portal vein, where they can directly interfere with hepatocytes (23). Our study results suggest an interesting interplay between gut microbiota richness and liver fat content. Currently there are no approved therapies for the treatment of NAFLD, therefore finding potential new targets (the gut microbiota) to treat NAFLD is desirable.

Since our combination treatment of DMR and GLP-1RA is offered as a package deal in this pilot study, we cannot identify the correlation of DMR or GLP-1RA individually with the gut microbiota. However, it is still interesting to speculate which of the two might be held largely responsible for the specific changes observed in the gut microbiota composition in the responders of the combination treatment. For GLP-1RA, there is no human data available of its influence on gut microbiota composition. In mice, treatment with liraglutide showed a trend toward increase in microbiota diversity, however this did not attain statistical significance (24). We therefore hypothesize that the here presented changes in gut microbiota diversity are mainly attributable due to DMR. However, the exact mechanism on how DMR influences the gut microbiota remains unknown and a causal relationship has yet to be established.

As we mentioned previously, this spin-off study has some limitations. First, it is an observational uncontrolled proof-of-concept study with a limited sample size. Second, we cannot determine the effect of each individual treatment component on glycaemia and microbiota diversity. Third, as we did not monitor dietary intake, we cannot rule out that changes in microbiota diversity occurred as an epiphenomenon during metabolic improvements. Fourth, we did find significant correlations between a change in HbA1c and a change in gut microbiota alpha diversity, and a change in liver fat and gut microbiota beta diversity, but these correlations were less clear by visual examination of the scatterplots. Larger randomized controlled studies are necessary to elucidate causal links between metabolic improvements, the gut microbiota and DMR.

In conclusion, we found that improved glycaemic control in patients with T2DM after initiation of DMR with GLP-1RA was correlated with an increase in gut microbiota richness. Additionally, a large change in liver fat content was correlated with a large change in gut microbiota diversity. This data supports the important role of the gut microbiota in metabolic diseases and its possible potential to modify disease. However, causality cannot be proven with this study.

## Data Availability Statement

Publicly available datasets were analyzed in this study. This data can be found here: [https://www.ebi.ac.uk/ena/browser/view/PRJEB51010?show=reads].

## Ethics Statement

The studies involving human participants were reviewed and approved by Ethics committee of the Amsterdam UMC, location AMC. The patients/participants provided their written informed consent to participate in this study.

## Author Contributions

Conceptualization, AB, FH, MS, MN, and JB. Investigation, SM and AB. Laboratory analyses, NS. Formal statistical analysis, SM, AB, and NS. Writing manuscript, SM and AB. Editing, AB, NS, FH, MN, and JB. Reviewing, AB, NS, FH, MS, MN, and JB. All authors contributed to the article and approved the submitted version.

## Funding

This study received funding from Fractyl Health Inc. The funder was not involved in the study design, collection, analysis, interpretation of data, the writing of this article or the decision to submit it for publication. MN is supported by a ZONMW VICI grant 2020 [09150182010020].

## Conflict of Interest

MN is in the Scientific Advisory Board of Caelus Pharmaceuticals, the Netherlands and Kaleido Biosciences, USA. However none of these are directly relevant to the current paper. FH reports speaker fees from Sanofi, Bioton, Astra Zeneca, and Boehringer Ingelheim. JB received research support from Fractyl Health Inc. for IRB-based studies and received a consultancy fee for a single advisory board meeting of Fractyl in September 2019.

The remaining authors declare that the research was conducted in the absence of any commercial or financial relationships that could be construed as a potential conflict of interest.

## Publisher’s Note

All claims expressed in this article are solely those of the authors and do not necessarily represent those of their affiliated organizations, or those of the publisher, the editors and the reviewers. Any product that may be evaluated in this article, or claim that may be made by its manufacturer, is not guaranteed or endorsed by the publisher.
