# Supplementary material for: A Changed Gut Microbiota Diversity Is Associated With Metabolic Improvements After Duodenal Mucosal Resurfacing With Glucagon-Like-Peptide-1 Receptor Agonist in Type 2 Diabetes in a Pilot Study
Source: Front Clin Diabetes Healthc. 2022 Jul 5;3:856661. doi: 10.3389/fcdhc.2022.856661 (PMC10012157; doi:10.3389/fcdhc.2022.856661)
Supplement: Supplementary file 1 [file DataSheet_1.zip › Data sheet 1/Supplementary table 1, Microbiome Manuscript.docx]

Supplementary table 1.

| **Inclusion Criteria:** | 1. Diagnosed with Type 2 Diabetes 2. 28 -75 years of age 3. Treatment with long acting insulin ≤ 5 years 4. On daily long acting insulin dose ≤ 1 U/kg 5. BMI ≥ 24 and ≤ 40 kg/m2 6. HbA1c ≤ 8.0% (64 mmol/mol) 7. Fasting C-peptide ≥ 0.5 nmol/L (1.5 ng/ml) 8. Willing to comply with study requirements and able to understand and comply with informed consent 9. Signed informed consent form |
| --- | --- |
| **Exclusion Criteria:** | 1. Diagnosed with Type 1 Diabetes or with a history of ketoacidosis 2. Fasting C-peptide < 0.5 nmol/L (1.5 ng/ml) 3. Current use of multiple daily doses insulin or insulin pump 4. Current use of a sulfonylurea derivate, GLP-1 analogue, DPP4 inhibitor, or meglitinide 5. A positive Anti-GAD test, as an indication of type 1 diabetes mellitus or Latent Autoimmune Diabetes of the Adult (LADA) with progressive beta-cell loss. 6. Previous GI surgery that could affect the ability to treat the duodenum such as subjects who have had a Bilroth 2, Roux-en-Y gastric bypass, or other similar procedures or conditions 7. History of chronic or acute pancreatitis 8. Known active hepatitis or active liver disease 9. Symptomatic gallstones or kidney stones, acute cholecystitis or history of duodenal inflammatory diseases including Crohn’s Disease and Celiac Disease 10. History of coagulopathy, upper gastro-intestinal bleeding conditions such as ulcers, gastric varices, strictures, congenital or acquired intestinal telangiectasia 11. Use of anticoagulation therapy (such as phenprocoumon and acenocoumarol) and novel oral anticoagulants (such as rivaroxaban, apixaban, edoxaban and dabigatran) which cannot be discontinued for 7 days before and 14 days after the procedure 12. Use of P2Y12 inhibitors (clopidogrel, pasugrel, ticagrelor) which cannot be discontinued for 14 days before and 14 days after the procedure. Use of aspirin is allowed. 13. Unable to discontinue NSAIDs (non-steroidal anti-inflammatory drugs) during treatment through 4 weeks post procedure phase 14. Taking corticosteroids or drugs known to affect GI motility (e.g. Metoclopramide) 15. Receiving weight loss medications such as Meridia, Xenical, or over the counter weight loss medications 16. Persistent Anemia, defined as Hgb < 10 g/dl 17. eGFR or MDRD < 30 ml/min/1.73m^2 18. Active systemic infection 19. Active malignancy within the last 5 years 20. Not potential candidates for surgery or general anesthesia 21. Active illicit substance abuse or alcoholism 22. Pregnancy or wish getting pregnant in next year 23. Participating in another ongoing clinical trial of an investigational drug or device 24. Any other mental or physical condition which, in the opinion of the Investigator, makes the subject a poor candidate for clinical trial participation |
