# Supplementary material for: A Changed Gut Microbiota Diversity Is Associated With Metabolic Improvements After Duodenal Mucosal Resurfacing With Glucagon-Like-Peptide-1 Receptor Agonist in Type 2 Diabetes in a Pilot Study
Source: Front Clin Diabetes Healthc. 2022 Jul 5;3:856661. doi: 10.3389/fcdhc.2022.856661 (PMC10012157; doi:10.3389/fcdhc.2022.856661)
Supplement: Supplementary file 1 [file DataSheet_1.zip › Data sheet 1/Supplementary table 2, Microbiome Manuscript.docx]

Supplementary table 2.

| Patient characteristics (N=16) | |
| --- | --- |
| Age [years] | 61 (55–67) |
| Male gender, n (%) | 10 (63%) |
| Duration of T2D [years] | 11 (8–15) |
| Weight [kg] | 87.8 (80.2–99.7) |
| BMI [kg/m^2^] | 28.8 (26.5–31.7) |
| HbA1c [%], [mmol/mol] | 7.5 (7.1–7.9), 58 (54-63) |
| Fasting plasma glucose [mmol/l] | 10.1 (8.9–12.0) |
| Fasting plasma insulin [pmol/l] | 104 (49–178) |
| C-peptide [nmol/l] | 0.63 (0.55–0.91) |
| HOMA-IR | 8.4 (4.3–12.0) |
| Glucose-lowering medication | |
| Mean number of daily units of insulin | 31 (16–47) |
| Insulin monotherapy, n (%) | 2 (12.5%) |
| Oral glucose lowering medications, n (%) | 14 (87.5%) |
| Metformin, n (%) | 13 (81.3%) |
| Empagliflozin, n (%) | 1 (6.25%) |
